# Supplementary figures and images for: Insights into Environmental Drivers on the Reproductive Cycle of Diopatra neapolitana (Polychaeta: Onuphidae)
Source: Biology (Basel). 2022 Oct 14;11(10):1504. doi: 10.3390/biology11101504 (PMC9598824; doi:10.3390/biology11101504)

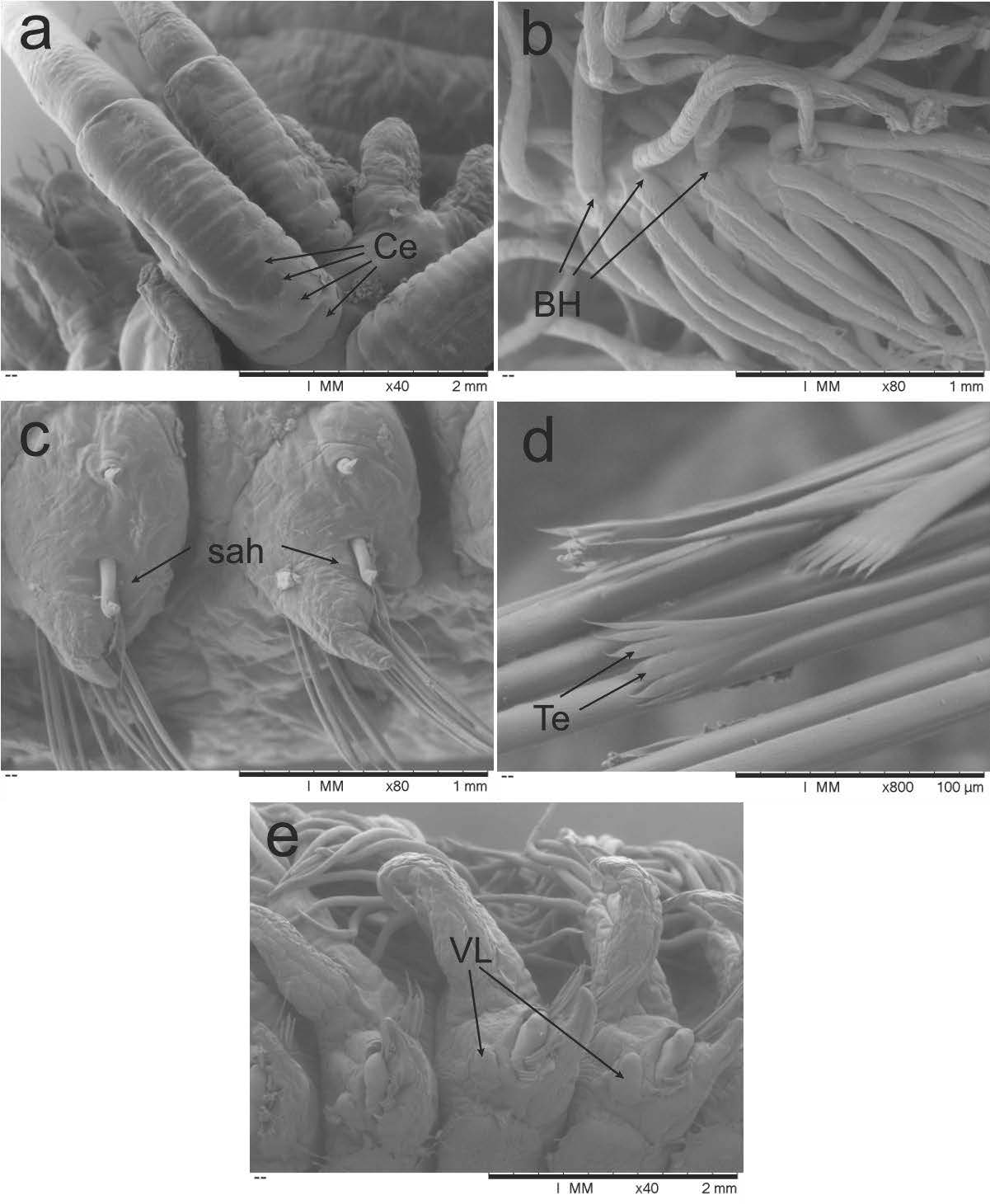

Supplement: Supplementary file 1 [file biology-11-01504-s001.zip › biology-1871489-Figure S1.jpg]
